# Differential effects of the cystic fibrosis lung inflammatory environment on mesenchymal stromal cells

**DOI:** 10.1152/ajplung.00218.2020

**Published:** 2020-09-09

**Authors:** Soraia C. Abreu, Thomas H. Hampton, Evan Hoffman, Jacob Dearborn, Alix Ashare, Karatatiwant Singh Sidhu, Dwight E. Matthews, David H. McKenna, Eyal Amiel, Jayita Barua, Anna Krasnodembskaya, Karen English, Bernard Mahon, Claudia Dos Santos, Fernanda F. Cruz, Daniel C. Chambers, Kathleen D. Liu, Michael A. Matthay, Robert A. Cramer, Bruce A. Stanton, Patricia R. M. Rocco, Matthew J. Wargo, Daniel J. Weiss, Sara Rolandsson Enes

**Affiliations:** ^1^Department of Medicine, Larner College of Medicine, University of Vermont, Burlington, Vermont; ^2^Laboratory of Pulmonary Investigation, Carlos Chagas Filho Institute of Biophysics, Federal University of Rio de Janeiro, Rio de Janeiro, Brazil; ^3^Department of Microbiology and Immunology, Geisel School of Medicine at Dartmouth, Hanover, New Hampshire; ^4^Section of Pulmonary and Critical Care Medicine, Dartmouth-Hitchcock Medical Center, Lebanon, New Hampshire; ^5^Department of Chemistry, University of Vermont, Burlington, Vermont; ^6^Department of Laboratory Medicine and Pathology, University of Minnesota, Minneapolis, Minnesota; ^7^Department of Biomedical and Health Sciences, College of Nursing and Health Sciences, University of Vermont, Burlington, Vermont; ^8^Division of Pulmonary Disease and Critical Care, University of Vermont, and The Vermont Lung Center, Burlington, Vermont; ^9^Wellcome-Wolfson Institute for Experimental Medicine, School of Medicine, Dentistry and Biomedical Sciences, Queen’s University of Belfast, Belfast, United Kingdom; ^10^Cellular Immunology Laboratory, Biology Department, Human Health Research Institute, Maynooth University, Maynooth, Ireland; ^11^Immunology & Cell Biology Laboratory, Biology Department, Human Health Research Institute, Maynooth University, Maynooth, Ireland; ^12^Departments of Medicine and Critical Care Medicine and the Keenan Research Center for Biomedical Science, St. Michael’s Hospital, University of Toronto, Toronto, Ontario, Canada; ^13^National Institute of Science and Technology for Regenerative Medicine, Rio de Janeiro, Brazil; ^14^School of Medicine, University of Queensland, Brisbane, Queensland, Australia; ^15^Queenland Lung Transplant Service, The Prince Charles Hospital, Brisbane, Queensland, Australia; ^16^Departments of Medicine and Anesthesiology and the Cardiovascular Research Institute, University of California, San Francisco, California; ^17^Department of Microbiology & Molecular Genetics, Larner College of Medicine, University of Vermont, Burlington, Vermont; ^18^Department of Experimental Medical Science, Lung Biology Unit, Lund University, Lund, Sweden

**Keywords:** *Aspergillus* infection, cell therapy, cystic fibrosis, gliotoxin, mesenchymal stromal cell

## Abstract

Growing evidence demonstrates that human mesenchymal stromal cells (MSCs) modify their in vivo anti-inflammatory actions depending on the specific inflammatory environment encountered. Understanding this better is crucial to refine MSC-based cell therapies for lung and other diseases. Using acute exacerbations of cystic fibrosis (CF) lung disease as a model, the effects of ex vivo MSC exposure to clinical bronchoalveolar lavage fluid (BALF) samples, as a surrogate for the in vivo clinical lung environment, on MSC viability, gene expression, secreted cytokines, and mitochondrial function were compared with effects of BALF collected from healthy volunteers. CF BALF samples that cultured positive for *Aspergillus sp.* (Asp) induced rapid MSC death, usually within several hours of exposure. Further analyses suggested the fungal toxin gliotoxin as a potential mediator contributing to CF BALF-induced MSC death. RNA sequencing analyses of MSCs exposed to either Asp+ or Asp− CF BALF samples identified a number of differentially expressed transcripts, including those involved in interferon signaling, antimicrobial gene expression, and cell death. Toxicity did not correlate with bacterial lung infections. These results suggest that the potential use of MSC-based cell therapies for CF or other lung diseases may not be warranted in the presence of *Aspergillus*.

## INTRODUCTION

Advances in cell-based therapies for lung diseases provide a platform for the development of new therapeutic approaches for acute lung diseases and possibly other chronic pulmonary conditions. Mesenchymal stromal cell (MSC)-based therapies have shown promise because of their immunomodulatory properties (4, 25). MSCs sense the inflammatory environment and respond by releasing specific sets of anti-inflammatory mediators (23, 25, 28, 39, 45). Notably, following systemic or local airway-based MSC administration in preclinical lung injury and critical illness models, many if not most injury end points are ameliorated (43, 48). However, whereas systemic MSC administration has proven safe in clinical investigations, there has been no clear efficacy in a spectrum of patients with lung disease studied to date, although these trials were primarily designed for safety and not well powered for efficacy (6, 30, 49).

Growing literature demonstrates that bronchoalveolar lavage fluid (BALF) or serum obtained from preclinical lung disease models or from patients with acute respiratory distress syndrome (ARDS) elicits distinct changes in MSC functions (1, 2, 5, 21, 32, 42). For example, ARDS BALF suppressed cytokine production, increased M2 macrophage marker expression, and augmented phagocytic capacity of human monocytes (32). Other recent data demonstrated that ARDS BALF altered MSC protein expression with correlative effects on potential protective actions in acute lung injury (21). Furthermore, we have demonstrated that human bone marrow-derived MSCs (hMSCs) exposed to BALF samples from patients with either ARDS or non-ARDS lung diseases displayed both common and disease-specific hMSC phenotypes, data that further support the hypothesis that different lung inflammatory environments have the potential to alter MSC behaviors (1).

The chronic lung inflammation in cystic fibrosis (CF) is also a potential target for MSC-based cell therapies, and an initial clinical investigation of safety following systemic administration of hMSCs in patients with CF (NCT02866721) is underway. However, the CF lung environment is highly inflammatory and is frequently colonized with fungi as well as bacteria. Thus, we postulated that the CF lung environment might adversely alter hMSC functions and efficacy. Using BALF samples as a surrogate for the in vivo lung environment, we explored how the inflammatory and infectious environment in CF lung disease alters hMSC functions.

## METHODS

### 

#### Human BALF.

BALF samples from patients with CF were obtained prospectively at the University of Vermont (UVM) Medical Center, the University of Colorado, and Dartmouth–Hitchcock Medical Center. Samples were obtained as part of normal clinical care of patients undergoing CF respiratory exacerbations. Normal volunteer (healthy control, HC) BALF was obtained from Dartmouth, University of Brisbane, and Queen’s University Belfast. All BALF samples were collected under appropriate IRB protocols, and samples were de-identified and numerically coded. Primary BALF samples collected at UVM were centrifuged at 500 g for 5 min to remove any remaining viable cells or cell debris. BALF samples obtained from Colorado, Dartmouth, Brisbane, and Belfast were comparably processed before shipping. Microbiological cultures for BALF samples from patients with CF were performed immediately after collection at each respective institution using standard routine protocols and obtained under appropriate institutional protocol and Health Insurance Portability and Accountability Act (HIPAA) guidelines. Delineation of each sample and how it was used is shown in Table 1. Individual rather than pooled samples were used for all analyses.

**Table 1.** Delineation of each sample and how it was used in this study

Sample No.	Location	*Aspergillus* status	Study
*1*	Dartmouth	na	Mass spectrometry (GT), RNA Seq, RT-PCR, LDH 1 h
*2*	Dartmouth	na	Mass spectrometry (GT), RNA Seq, RT-PCR, LDH 1 h, Luminex BALF
*3*	Dartmouth	na	Mass spectrometry (GT), RNA Seq, RT-PCR, LDH 1 h, Luminex BALF
*4*	Dartmouth	na	Mass spectrometry (GT), RNA Seq, LDH 1 h, Luminex BALF
*5*	Dartmouth	na	Mass spectrometry (GT), RNA Seq, RT-PCR, LDH 1 h, Luminex BALF
*6*	Dartmouth	na	Mass spectrometry (GT), RNA Seq, RT-PCR, LDH 1 h, Luminex BALF
*7*	Dartmouth	na	Mass spectrometry (GT)
*8*	Dartmouth	na	Mass spectrometry (GT)
*9*	Dartmouth	na	Mass spectrometry (GT), Luminex BALF
*10*	Dartmouth	na	Mass spectrometry (GT), Luminex BALF
*11*	Dartmouth	na	Mass spectrometry (GT)
*12*	Dartmouth	na	Mass spectrometry (GT)
*13*	Dartmouth	na	Mass spectrometry (GT)
*14*	Dartmouth	na	Mass spectrometry (GT)
*15*	Dartmouth	na	Mass spectrometry (GT)
*16*	Australia	na	Osmolality, dsDNA, protein, protease
*17*	Australia	na	Osmolality, histones, dsDNA, protein, protease
*18*	Australia	na	Luminex CM, LDH 1 h, LDH 5 h, osmolality, dsDNA, protein, protease
*19*	Australia	na	Osmolality, histones, dsDNA, protein, protease
*20*	Australia	na	Luminex CM, LDH 1 h, LDH 5 h, osmolality, protein
*21*	Australia	na	Osmolality, protein
*22*	Australia	na	Luminex CM, LDH 1 h, LDH 5 h, osmolality, dsDNA, protein, protease
*23*	Australia	na	Osmolality, histones, dsDNA, protein, protease
*24*	Australia	na	Osmolality, dsDNA, protein, protease
*25*	Australia	na	Osmolality, dsDNA, protein, protease
*26*	Australia	na	Osmolality, histones, dsDNA, protein, protease
*27*	Australia	na	LDH 1 h, LDH 5 h, osmolality, protein
*28*	Australia	na	Osmolality, protein, protease
*29*	Australia	na	Osmolality, histones, dsDNA, protein, protease
*30*	Australia	na	Osmolality, dsDNA, protein, protease
*31*	Australia	na	Osmolality, histones, dsDNA, protein, protease
*32*	Dartmouth	neg	Mass spectrometry (GT), boiled BALF, RNA Seq, RT-PCR, LDH 1 h
*33*	Dartmouth	neg	Mass spectrometry (GT), RNA Seq, LDH 1 h
*34*	Dartmouth	neg	Mass spectrometry (GT), RT-PCR, LDH 1 h
*35*	Dartmouth	pos	Mass spectrometry (GT), RNA Seq, LDH 1 h
*36*	Dartmouth	pos	Mass spectrometry (GT), RNA Seq, RT-PCR, LDH 1 h
*37*	Dartmouth	pos	Mass spectrometry (GT), RNA Seq, RT-PCR, LDH 1 h
*38*	UVMMC	neg	Osmolality, dsDNA, protein, protease
*39*	UVMMC	neg	Osmolality, dsDNA, protein, protease
*40*	UVMMC	pos	Osmolality, histones, dsDNA, protein, protease
*41*	UVMMC	pos	Osmolality, histones, dsDNA, protein, protease
*42*	UVMMC	pos	Protein
*43*	UVMMC	pos	Osmolality, histones, dsDNA, protein, protease
*44*	UVMMC	neg	LDH 1 h, LDH 5 h
*45*	UVMMC	pos	Mass spectrometry (GT), osmolality, histones, dsDNA, protease
*46*	UVMMC	pos	Mass spectrometry (GT), osmolality, histones, dsDNA, protein, protease
*47*	UVMMC	neg	Luminex CM, RNA Seq, RT-PCR, LDH 1 h, LDH 5 h, osmolality, dsDNA, protein, protease
*48*	UVMMC	pos	Mass spectrometry (GT), Luminex CM, Luminex BALF, RNA Seq, RT-PCR, LDH 1 h, LDH 5 h, osmolality, histones, dsDNA, protein, protease
*49*	UVMMC	pos	LDH 1 h, LDH 5 h, osmolality, histones, mass spectrometry (GT), Luminex BALF, dsDNA, protein, protease, Luminex CM, RNA Seq, RT-PCR
*50*	UVMMC	neg	Luminex CM, LDH 1 h, LDH 5 h, osmolality, histones, dsDNA, protein
*51*	UVMMC	neg	Luminex CM, LDH 1 h, LDH 5 h, osmolality, protein
*52*	UVMMC	pos	Mass spectrometry (GT), Luminex CM, Luminex BALF, LDH 1 h, LDH 5 h, osmolality, histones, dsDNA, protein
*53*	UVMMC	pos	Mass spectrometry (GT), DTT BALF, Luminex CM, Luminex BALF, HLA, LDH 1 h, LDH 5 h, osmolality, histones, dsDNA, protein
*54*	UVMMC	neg	Luminex CM, Luminex BALF, HLA, LDH 1 h, LDH 5 h, osmolality, histones, dsDNA, protein
*55*	UVMMC	pos	Mass spectrometry (GT), Luminex CM, HLA, LDH 1 h, LDH 5 h, osmolality, histones, dsDNA, protein, Luminex BALF
*56*	UVMMC	neg	Luminex CM, LDH 1 h, LDH 5 h, osmolality, dsDNA
*57*	UVMMC	neg	Mass spectrometry (GT), Luminex CM, Luminex BALF, HLA, LDH 1 h, LDH 5 h, osmolality, histones, dsDNA, protein
*58*	UVMMC	pos	Boiled BALF, DTT BALF, Luminex BALF, HLA, RNA Seq, RT-PCR, osmolality, histones, dsDNA, protein
*59*	UVMMC	neg	Mass spectrometry (GT), Luminex BALF, HLA, osmolality, histones, dsDNA, protein
*60*	UVMMC	neg	Mass spectrometry (GT), RNA Seq, RT-PCR
*61*	UVMMC	neg	Mass spectrometry (GT), boiled BALF, RNA Seq, RT-PCR
*62*	UVMMC	neg	Mass spectrometry (GT)
*63*	UVMMC	neg	Mass spectrometry (GT)
*64*	UVMMC	neg	Mass spectrometry (GT), boiled BALF
*65*	Colorado	neg	Mass spectrometry (GT)
*66*	Colorado	neg	Mass spectrometry (GT)
*67*	Colorado	neg	Mass spectrometry (GT)
*68*	Colorado	neg	Mass spectrometry (GT)
*69*	Colorado	neg	Mass spectrometry (GT)
*70*	Colorado	neg	Mass spectrometry (GT)
*71*	Colorado	neg	Mass spectrometry (GT)
*72*	Colorado	neg	Mass spectrometry (GT)
*73*	Colorado	neg	Mass spectrometry (GT)
*74*	Colorado	neg	Mass spectrometry (GT)
*75*	Colorado	neg	Mass spectrometry (GT)
*76*	Colorado	neg	Mass spectrometry (GT)
*77*	Colorado	neg	Mass spectrometry (GT)
*78*	Colorado	neg	Mass spectrometry (GT), boiled BALF
*79*	Colorado	neg	Mass spectrometry (GT), boiled BALF
*80*	Colorado	pos	Mass spectrometry (GT)
*81*	Colorado	pos	Mass spectrometry (GT)
*82*	Colorado	pos	Mass spectrometry (GT), boiled BALF, DTT BALF
*83*	Colorado	pos	Mass spectrometry (GT)
*84*	Colorado	pos	Mass spectrometry (GT)
*85*	Colorado	pos	Mass spectrometry (GT), boiled BALF, DTT BALF
*86*	Colorado	pos	Mass spectrometry (GT)
*87*	Colorado	pos	Mass spectrometry (GT)
*88*	Colorado	pos	Mass spectrometry (GT), boiled BALF
*89*	Colorado	pos	Mass spectrometry (GT)
*90*	Colorado	pos	Mass spectrometry (GT)
*91*	Colorado	pos	Mass spectrometry (GT)
*92*	Colorado	pos	Mass spectrometry (GT), boiled BALF, DTT BALF
*93*	Colorado	pos	Mass spectrometry (GT), boiled BALF, DTT BALF
*94*	Colorado	pos	Mass spectrometry (GT)

Asp+, *Aspergillus* positive; Asp−, *Aspergillus* negative; BALF, bronchoalveolar lavage fluid; CF, cystic fibrosis; CM, conditioned media; Dartmouth, Dartmouth Hitchcock Medical Center; DTT, dithiothreitol; GT, gliotoxin; HC, healthy control subject; HLA, human leukocyte antigen; LDH, lactate dehydrogenase; na, not available; neg, negative; RNA Seq, RNA sequencing; UVMMC, University of Vermont Medical Center.

Protease activity in BALF samples was assessed by using the Pierce Fluorescence Protease Assay Kit (Thermo Scientific, Cat. No. 23266) according to the manufacturer’s instructions. Briefly, 100 µL of BALF samples obtained from patients with CF (*n* = 10), HC (*n* = 13), or standards was incubated with 100 µL of FTC-casein working reagent at room temperature. Following incubation, fluorescence was measured using a spectrophotometer plate reader (excitation: 485/20, emission: 528/20). All samples were analyzed in duplicate. BALF osmolality was measured using a vapor pressure osmometer (Vapo) according to the manufacturer’s instructions. Briefly, 10 µL of BALF samples obtained from patients with CF (*n* = 20) and HC (*n* = 16) was loaded to a sample disk in the sample holder and osmolality (mmol/kg) was analyzed. The total amount of BALF dsDNA content was measured in patients with CF (*n* = 19) and HC (*n* = 12) using the Qubit Fluorometer dsDNA HS Assay Kit (10 pg/µL – 100 ng/µL). Total BALF protein was measured in patients with CF (*n* = 19) and HC (*n* = 16) by using the Pierce BCA Protein Assay Kit (Thermo Scientific, Cat. No. 23225) according to the manufacturer’s instructions. Absorbance was measured at 562 nm by using a spectrophotometer plate reader. All samples were analyzed in duplicate. BALF histone-complex DNA fragments were measured in patients with CF (*n* = 15) and HC (*n* = 6) by using the Cell Death Detection ELISA Kit (Roche) according to the manufacturer’s instructions. Samples were analyzed in duplicate.

#### In vitro exposure of hMSCs.

Human MSCs (hMSCs) were obtained from the National Heart, Lung, and Blood Institute’s Production Assistance for Cellular Therapies (PACT) program (University of Minnesota) (35) and routinely cultured in minimum essential medium/Earle’s balanced salt solution (MEM/EBSS) media (HyClone) supplemented with 20% fetal bovine serum (HyClone) and 1% penicillin/streptomycin in standard tissue culture incubators. The hMSCs used in this study were obtained from multiple healthy volunteers and have previously been used in similar studies (1). Cells at passage 3–6 were used for experiments. For exposures, hMSCs were seeded into six-well plates (200,000 cells/well) in normal growth media and allowed to attach overnight. Following adherence, cells were synchronized for 24 h in serum-free media (MEM/EBSS) (1). The hMSCs were then exposed to serum-free media containing BALF (20% volume:volume), *Aspergillus fumigatus* filtrate (wild type, WT, 1:1), null mutant ΔgliP filtrate (1:1), or pure gliotoxin (100, 500, or 1,000 ng/mL) for 1 or 5 h. The BALF dilutions and time of exposures were determined by our previous studies exposing hMSCs to clinical BALF samples (1). Serum-free media were added to control cells (unstimulated). Following incubations, supernatants were removed by aspiration and fresh serum-free media were added. After 24 h, conditioned media were collected for different analyses and cells were lysed with TRIzol reagent for RNA sequencing and other analyses. For gliotoxin neutralization experiments, samples were heat-treated (≥92.4°C) for 20 min or treated with 100 µM dithiothreitol (DTT) before exposures. Untreated BALF samples were used as controls for the neutralization experiments.

#### Cytotoxicity.

hMSC viability was assessed using a Pierce LDH Cytotoxicity Assay Kit (Thermo Scientific) according to the manufacturer’s instructions. An AMG EVOS Cell Imaging System was used to obtain photomicrographs of cell following exposures to assess qualitative appearance of the cells.

#### Mitochondrial respiration.

hMSCs were cultured as described above in *In vitro exposure of hMSCs*. On the day before metabolic flux analysis, hMSCs were seeded at a density of 25 × 10^3^, 50 × 10^3^, and 75 × 10^3^ cells/well in an XF-96^e^ cell culture plate (Agilent/Seahorse Bioscience). XF-96^e^ probe plates were hydrated and prepared according to the manufacturer’s instructions (Agilent/Seahorse Bioscience). On the day of the assay, hMSC culture medium was replaced with 180 µL of “XF running media” (XF phenol red-free, glucose-free DMEM, supplemented with FBS, 2 mM L-glutamine, and 5 mM glucose). The XF-96^e^ probe plate was injected with 20 µL of a 10-fold concentrate of indicated treatments (media, DTT, GT, or GT + DTT) in one port, and 1 µM Rotenone/Antimycin-A in a second port. For the data acquisition protocol, three baseline reads were recorded, followed by injection of indicated treatment, 1 h of additional reads, followed by the second injection of rotenone/antimycin-A, and followed by three final reads. Data were normalized to the average of the three initial baseline reads to represent a percent fluctuation from pretreatment respiration levels. “Percent basal respiration sensitive to treatment” was calculated as the difference between the basal respiration rate and the respiration rate immediately following treatment injection. “Residual respiration rate after treatment” was calculated as the difference between the posttreatment respiration rate and the average respiration rate following the rotenone/antimycin-A injection.

#### Luminex analyses.

Both BALF samples and conditioned media obtained from BALF or control-exposed (serum-free media) hMSCs were analyzed using a human Luminex Assay Kit (R&D Systems). All samples were diluted 1:2 and analyzed using the Bio-Rad Bioplex Analyzer in duplicate according to the manufacturer’s instructions. Analytes included A disintegrin and metalloproteinase with thrombospondin motifs 13 (ADAMTS13), chemokine (C-X-C motif) ligand 8/interleukin 8 (CXCL8/IL-8), sFASL (soluble FAS ligand), granulocyte-macrophage colony-stimulating factor (GM-CSF), IFN-γ, IL-10, IL-13, IL-2, IL-4, leptin, macrophage migration inhibitory factor (MIF), macrophage inflammatory protein 1β (CCL4/MIP-1β), osteopontin, TNF-α, CD44, FAS, G-CSF, HGF, IL-1β, IL-12 p70, IL-18, IL-36β/IL-1F8, IL-6, monocyte chemoattractant protein 1 (CCL2/MCP-1), macrophage inflammatory protein 1α (CCL3/MIP-1α), matrix metalloproteinase-3 (MMP3), and surfactant protein D (SP-D). Extrapolated values are presented as means ± SD, and values out of range (below) were set to 1.0.

#### Mass spectrometry.

To measure gliotoxin concentrations in BALF samples obtained from patients with CF (Asp+: *n* = 25, Asp−: *n* = 26) and HC subjects (HC: *n* = 15), gliotoxin was first extracted by adding 500-µL aliquots of 1:1 ethyl acetate plus ethyl ether at room temperature to 20-µL aliquots of the clinical samples and sonicated for 30 min without heat. Following sonication, samples were centrifuged at 15,000 *g *for 15 min at 4°C. The organic phase was extracted and evaporated, and the residue was reconstituted in 50 µL of 1:1 acetonitrile plus water. Standards of gliotoxin (Sigma-Aldrich) were prepared in a similar manner. Gliotoxin was then quantified by selected reaction monitoring (SRM) liquid chromatography-mass spectrometry (LC/MS) using a Shimadzu SIL-20A HPLC and ABI-SCIEX 4000 QTrap triple quadrupole mass spectrometer using the Atlantis dC18 HPLC column (1 mm × 150 mm, 5-um particle). Mobile phase A was 0.1% formic acid in LC/MS grade water, and mobile phase B was 0.1% formic acid in acetonitrile. A linear gradient was run from 25% to 98% mobile phase B over 12 min, held for 5 min, and then reversed to original composition of 25% mobile phase B over 8 min. The mobile phase flow was 80 µL/min, and the injection volume was 1.0 µL. The gliotoxin molecular ion [M+H]+  = 327.0 was selected and fragmented, and the fragment ion m/z = 263.1 was monitored for quantification. Using the calibration curve obtained from the gliotoxin standards, the gliotoxin content was measured for each sample analyzed. Limit of detection was 50 ng/mL.

#### Flow cytometry.

Cultured cells were harvested after exposure to BALF samples for 5 h (*n* = 6), nonspecific binding was blocked (FC block, BD Pharmingen, Cat. No. 564220), and cells were stained with the directly conjugated antibodies HLA-ABC (BioLegend, Cat No. 311404, clone: W6/32) and HLA-DR (BioLegend, Cat. No. 307636, clone: L243), as previously described (38). Unstimulated hMSCs were used as controls. Dead cells were excluded using live/dead staining (Invitrogen, Cat No. L23105), and doublets were excluded by gating on side scatter-height (SSC-H) versus side scatter-width (SSC-W) and forward scatter-height (FSC-H) versus forward scatter-width (FSC-W). Samples were analyzed on a BD LSRII using BD FACSDiva v8 software. Data analysis was performed using FlowJo software v.10. Unstimulated hMSCs were used as controls (*n* = 3).

#### RNA isolation and sequencing analysis.

Total RNA was extracted from unstimulated hMSCs (*n* = 5) and hMSCs exposed to HC BALF (*n* = 6), CF Asp+ BALF (*n* = 6), or CF Asp− BALF (*n* = 5) for 1 h using standard TRIzol extraction protocol. Briefly, hMSCs were lysed with TRIzol reagent directly in the cell culture dish, samples were phase-separated using chloroform, and RNA was isolated using 100% isopropanol, followed by a 75% ethanol RNA wash. RNA pellets were allowed to air-dry and resuspended in RNase-free water. RNA was further cleaned using RNeasy spin columns according to the manufacturer’s instructions. RNA amount and quality were measured using NanoDrop One (Thermo Scientific). For RT-PCR, cDNA was synthesized by using the iScript cDNA Synthesis Kit (Bio-Rad) according to the manufacturer’s instructions. All RT-PCR analyses were performed using iQ SYBR Green Supermix (Bio-Rad) according to the manufacturer’s instructions on a CFX96 Real-Time System (Bio-Rad). List of RT-PCR primer sequences is available upon request.

Sequencing was performed by the Genomics Shared Resource at the Geisel School of Medicine at Dartmouth University. RNA was quantified on a Qubit fluorometer, and quality was assessed on a Fragment Analyzer instrument (Agilent). All RNAs with RNA quality number (RQN) values > 6 were processed for RNA sequencing. Libraries were prepared using the Kapa mRNA HyperPrep Kit using 100 ng of total RNA as input, according to the manufacturer’s protocol, multiplexed onto a single NextSeq 500 High Output run, and sequenced to a minimum depth of 15 M single-end 75-bp reads per sample. Reads were aligned to the human genome assembly GRCh37.5 downloaded from GenBank in CLC Genomics Workbench (Qiagen, Germantown, MD), and count tables of reads aligned to each gene were exported for further analysis with the R software environment for statistical computing and graphics (34). Count tables of reads aligned to each gene were analyzed using edgeR (31, 36). Only genes with at least one count in each sample were included in further analysis. Libraries were normalized in edgeR using the trimmed mean of M-values (TMM). Tagwise dispersion for generalized linear models (GLMs) based on Cox–Reid (CR) profile-adjusted likelihood method was used to estimate differential gene expression between various types of BALF exposure.

#### Statistical analyses.

All data are presented as means ± SD, unless otherwise stated. Mann–Whitney tests, paired t**tests, or Fisher’s exact tests were used to assess differences between two groups. Kruskal–Wallis tests (Dunn’s post hoc test) or one-way ANOVA (Tukey post hoc test) was used to assess differences between three or more groups. Correlations between hMSC deaths by CF BALF components were tested using Spearman correlation tests. Linear models were used to assess significance of relationships between gliotoxin concentration and cell survival. Statistical analyses were performed using GraphPad Prism software, R, or edgeR. edgeR was used to normalize sequence counts based on differing library sizes and assess differential gene expression differences between unstimulated, HC, Asp+, and Asp− samples to identify sets of differentially expressed genes. Hierarchical clustering was performed using gplots, and GGally was used to visualize correlations between effects identified by edgeR. Venn diagrams were produced with gplots. Colored line graphs were produced using ggplot2 and direct labels. P values ≤0.05 were considered as significant, except in the case of RNA sequencing data analyzed in edgeR, where multiple hypothesis-corrected false discovery rates (FDRs) less than 0.05 were considered significant.

## RESULTS

### 

#### Exposure to BALF obtained from a subpopulation of patients with CF-induced hMSC death.

**Fig. 1.**
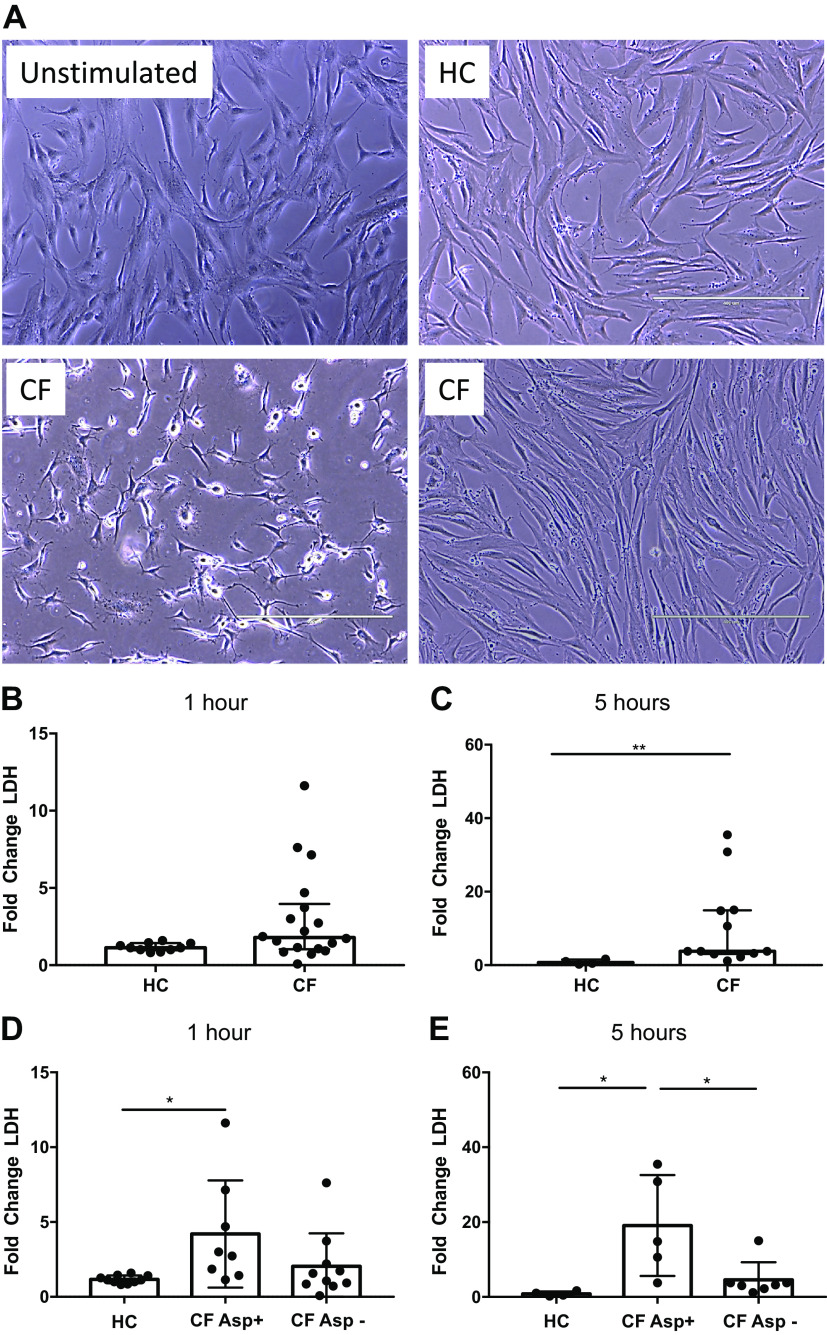
A subpopulation of clinical CF BALF samples provokes hMSC death. Representative phase contrast photomicrographs (×10) of hMSCs exposed for 5 h to 20% BALF samples obtained from patients with CF or from healthy volunteers (healthy control, HC). Unstimulated hMSCs were used as control. Representative images of exposures to two different CF BALF samples are depicted, one which stimulated cell death (lower left) and one which had no obvious effect (lower right) (*A*). Cytotoxicity was evaluated in conditioned medium using a standard LDH assay following either 1 h (HC: *n* = 10 and CF: *n* = 18) (*B*) or 5 h (HC: *n* = 4 and CF: *n* = 12) (*C*) exposure. Data are presented as median with interquartile range of fold change (stimulated sample/unstimulated control) with statistical analyses performed with the nonparametric Mann–Whitney test. The LDH data from (*B *and *C*) were reanalyzed and regrouped depending on the *Aspergillus* status (Asp+ or Asp−) of the CF BALF samples. LDH release after 1 h of exposure (HC: *n* = 10, Asp+: *n* = 8, and Asp−: *n* = 10) (*D*). LDH release after 5 h of exposure (HC: *n* = 4, Asp+: *n* = 5, and Asp−: *n* = 7) (*E*). Data are presented as means ± SD of fold change (stimulated sample/unstimulated control), and statistical analysis was performed by one-way ANOVA followed by Tukey’s post hoc test. Scale bar represents 400 µm. Asp+, *Aspergillus* positive; Asp−, *Aspergillus* negative; BALF, bronchoalveolar lavage fluid; CF, cystic fibrosis; HC, healthy volunteers; hMSC, human bone marrow-derived mesenchymal stromal cell; LDH, lactate dehydrogenase; *P ≤ 0.05 and **P ≤ 0.01. Photomicrographs have been brightness-/contrast-adjusted.

**Fig. 2.**
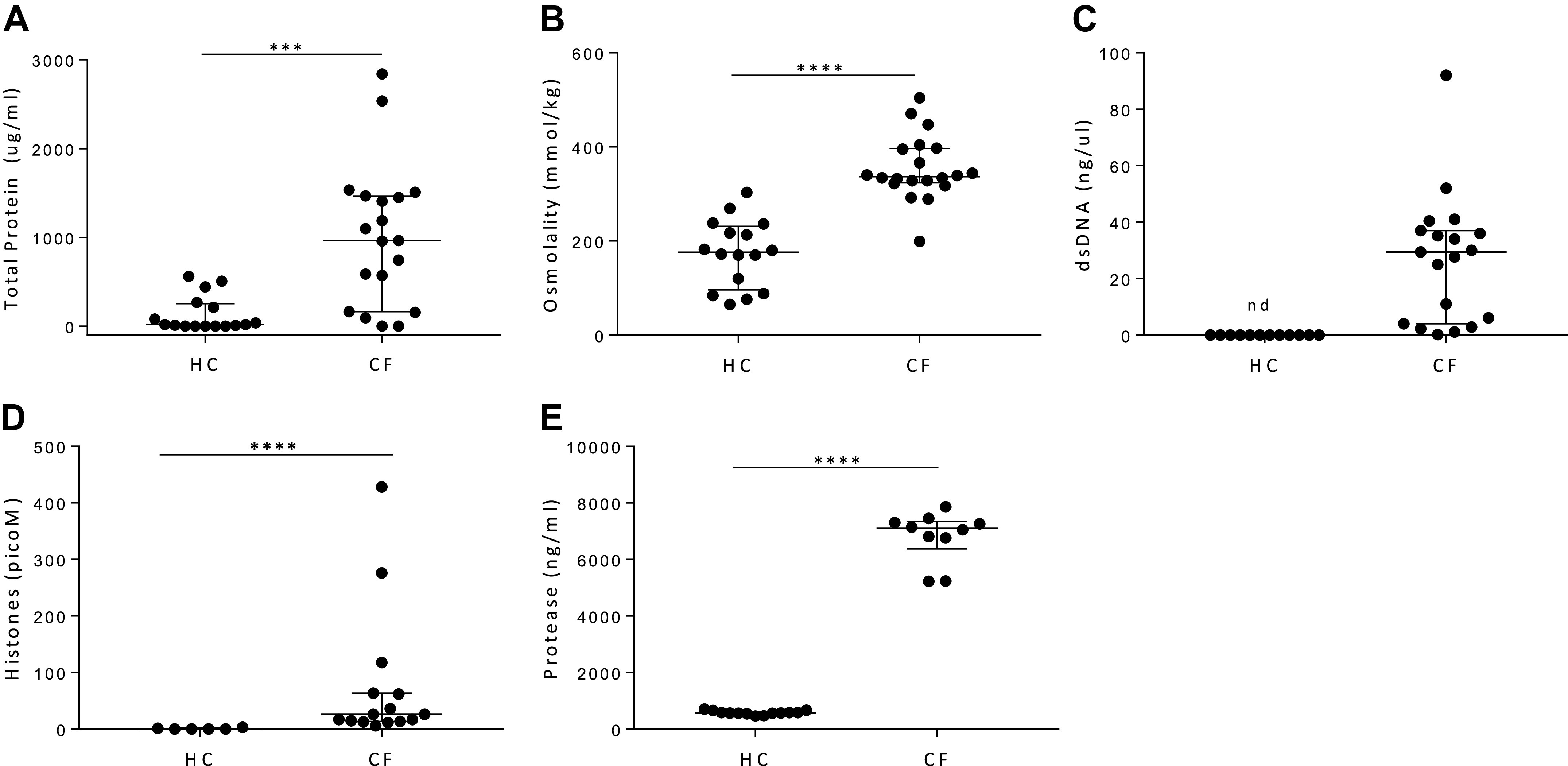
Although there are significant differences in content of healthy control versus CF BALF samples, none correlated with hMSC LDH release. To assess what responsible factors might be present in CF BALF samples, total protein (HC: *n* = 16 and CF: *n* = 19) (*A*), osmolality (HC: *n* = 16 and CF: *n* = 20) (*B*), dsDNA content (HC: *n* = 12 and CF: *n* = 19) (*C*), histone content (HC: *n* = 6 and CF: *n* = 15) (*D*), and protease activity (HC: *n* = 13 and CF: *n* = 10) (*E*) were assessed and compared with those found in BALF obtained from normal healthy volunteers. Data are presented as median with interquartile range, and statistical analysis was performed by nonparametric Mann–Whitney test. BALF, bronchoalveolar lavage fluid; CF, cystic fibrosis; HC, healthy volunteers; hMSC, human bone marrow-derived mesenchymal stromal cell; LDH, lactate dehydrogenase; nd, not detected; ***P ≤ 0.001 and ****P < 0.001.

To assess how the inflammatory environment present in CF lungs alters hMSCs, hMSCs were exposed for 1 or 5 h to BALF samples obtained from either patients with CF or HC subjects. Notably, hMSCs exposed to BALF samples from some, but not all, patients with CF resulted in increased cell death, as assessed by visual appearance (Fig. 1*A*) and by lactate dehydrogenase (LDH) release (Fig. 1, *B *and *C*). hMSCs exposed to CF BALF samples for 1 h resulted in increased cytotoxicity (LDH release) compared with hMSCs exposed to HC BALF samples (*P* = 0.056) (Fig. 1*B*). The observed LDH release further increased over time, reaching statistical significance after 5 h of exposure (*P* = 0.002) (Fig. 1*C*). Importantly, none of the healthy BALF samples killed the hMSCs, suggesting specific toxicity in the BALF environment from some of the patients with CF. To further examine differential hMSC responses to CF versus HC BALF and to ascertain potential links to cell death, conditioned media collected after BALF exposure were assessed for a broad panel of pro- and anti-inflammatory cytokines (Tables 2 and 3); however, none of the observed differences in cytokine secretion correlated with LDH release. We next attempted to determine what other components/properties in the CF BALF samples might be responsible for hMSC death, including total protein, osmolality, dsDNA content, histone concentration, protease activity, and levels of pro- and anti-inflammatory cytokines (Fig. 2; Table 4**)**. However, none of these parameters strongly correlated with the induction of hMSC death as determined by LDH release (Tables 4 and 5). Normalizing BALF cytokines to BALF total protein for each individual sample also did not result in significant correlations with hMSC death.

**Table 2.** Cytokines detected in CM (1 h)

	Uns	HC	CF
Cytokine (pg/mL)	Means (SD)	Means (SD)	Means (SD)
IL-8	1,221 (1,675)	71.5 (29.6)	262 (369)
IFN-γ	0 (0)	1.5 (2.6)	6.4 (12.3)
IL-2	0 (0)	37.2 (64.5)	29.1 (51.2)
MIF	394.1 (378.5)	29.8 (51.56)	4,956 (4,358)
Osteopontin	3,889 (1,475)	5,657 (2,020)	607.8 (1,464)
CD44	146.6 (185.6)	47.7 (56.4)	118.4 (319.8)
FAS	0 (0)	0 (0)	265.7 (195.6)
HGF	7.4 (10.4)	0 (0)	56.7 (53.4)
IL-18	0 (0)	66.6 (115.4)	47.5 (112.3)
IL-6	1,171 (1,425)	81.0 (69.0)	160 (294.5)
CCL2	1,950 (521.4)	1,595 (335.5)	309.3 (415.3)
CCL3	0 (0)	84.3 (145.9)	116.8 (142)
MMP3	1,864 (2,636)	0 (0)	9.3 (30.9)

Cytokines detected in hMSC cultures after 1-h BALF stimulation [HC (*n* = 3) or CF (*n* = 11)] or Uns (*n* = 2) using 27-plex Luminex assay. Means ± SD of extrapolated values are presented. Values out of range below were set to 1.0. ADAMTS13, sFASL (soluble FAS ligand), GM-CSF, IL-10, IL-13, IL-4, leptin, CCL4, TNF-α, G-CSF, IL-1β, IL-12, IL-36, and SP-D all had values out of range below for all groups. BALF, bronchoalveolar lavage fluid; CM, conditioned medium; CF, cystic fibrosis; HC, healthy control; hMSC, human bone marrow-derived mesenchymal stromal cell; Uns, unstimulated control cells.

**Table 3.** Cytokines detected in CM (5 h)

	Uns	HC	CF
Cytokine (pg/mL)	Means (SD)	Means (SD)	Means (SD)
IL-8	531 (530)	39.6 (24.3)	249 (290)
IL-2	102.1 (102)	1.0 (0.0)	33.8 (70.7)
MIF	1.0 (0.0)	1.0 (0.0)	10,168 (11,312)
Osteopontin	3,652 (806)	4,295 (648)	461 (1,526)
CD44	1.0 (0.0)	1.0 (0.0)	171 (435)
FAS	1.0 (0.0)	1.0 (0.0)	681 (173)
HGF	1.0 (0.0)	1.0 (0.0)	115 (67.4)
IL-18	185 (184)	1.0 (0.0)	7.11 (20.3)
IL-6	30.7 (0)	20.7 (18.5)	63.5 (101)
CCL2	914 (127)	1,252 (165)	193 (321)
CCL3	218 (217)	1.0 (0.0)	29.4 (89.7)

Cytokines detected in hMSC cultures after 5-h BALF stimulation [HC (*n* = 3) or CF (*n* = 11)] or Uns (*n* = 2) using 27-plex Luminex assay. Means ± SD of extrapolated values are presented. Values out of range below were set to 1.0. ADAMTS13, sFASL (soluble FAS ligand), GM-CSF, IL-10, IL-13, IFN-γ, IL-4, leptin, CCL4, TNF-α, G-CSF, IL-1β, IL-12, IL-36 beta, MMP3, and SP-D all had values out of range below for all groups. BALF, bronchoalveolar lavage fluid; CF, cystic fibrosis; CM, conditioned medium; HC, healthy control; hMSC, human bone marrow-derived mesenchymal stromal cell; Uns, unstimulated control cells.

**Table 4.** Cytokines detected in BALF from patients with CF

	Cytokines in BALF (pg/mL)			Correlations (CF BALF vs. LDH in CM)			
	CF BALF	HC BALF	Mann–Whitney	CF BALF versus LDH CM 1 h		CF BALF versus LDH CM 5 h	
	Means (SD)	Means (SD)	*P* value	Spearman r	*P* value	Spearman r	*P* value
ADAMTS13	9,168 (18,591)	1.00 (0.00)	0.475	−0.223	0.667	−0.535	0.238
CXCL8/IL-8	4,888 (2,572)	88.4 (90.7)	0.0002	−0.321	0.498	−0.429	0.354
sFASL (soluble FAS ligand)	80.43 (86.40)	1.12 (0.35)	0.0003	−0.821	0.034	−0.643	0.139
GM-CSF	1.04 (0.11)	2.63 (4.52)	>0.999	−0.408	0.574	−0.612	0.286
IL-13	1,184 (488)	52.3 (136)	0.002	−0.214	0.662	−0.321	0.498
IL-2	88.4 (151)	1.00 (0.00)	0.089	−0.571	0.2	−0.493	0.257
IL-4	91.1 (33.7)	23.4 (14.5)	0.001	−0.324	0.482	−0.505	0.258
MIF	6,294 (6,225)	16,404 (10,262)	0.055	−0.25	0.595	−0.429	0.354
CCL4/MIP-1 beta	1,110 (718)	68.1 (118)	0.002	−0.714	0.088	−0.429	0.354
Osteopontin	7,540 (7,972)	5,317 (602)	0.837	−0.643	0.139	−0.357	0.444
TNF-alpha	30.50 (63.3)	1.00 (0.00)	0.006	−0.321	0.498	−0.357	0.444
CD44	1,144 (2,025)	330 (238)	0.174	−0.429	0.354	−0.286	0.556
FAS	16,058 (13,948)	150 (110)	0.017	−0.75	0.066	−0.643	0.139
G-CSF	124 (122)	51.0 (61.1)	0.09	0.714	0.088	0.75	0.066
HGF	908 (1,144)	20.73 (11.0)	0.005	−0.429	0.354	−0.286	0.556
IL-1 beta	3,758 (5,692)	11.38 (9.7)	0.002	−0.143	0.783	−0.036	0.964
IL-12 p70	81.4 (57.2)	1.00 (0.00)	0.006	0.071	0.906	0.107	0.84
IL-18	121 (94.6)	39.64 (36.6)	0.112	0.185	0.719	0.074	0.893
IL-36 beta/IL-1F8	6.47 (4.99)	5.03 (3.78)	0.452	−0.107	0.84	−0.071	0.906
IL-6	48.4 (71.4)	1.56 (1.09)	0.0003	−0.107	0.84	0	>0.999
CCL2/MCP-1	293 (318)	52.41 (35.3)	0.021	−0.179	0.713	0.036	0.964
CCL3 MIP-1 alpha	263 (265)	87.94 (72.6)	0.326	−0.519	0.245	−0.259	0.591
MMP-3	114 (156)	8.97 (15.7)	0.005	−0.071	0.906	0.214	0.662
SP-D	15,764 (35,016)	72,854 (35,018)	0.005	−0.071	0.906	0.179	0.713

Cytokines detected in clinical CF BALF samples (*n* = 9) and healthy control subjects (*n* = 7) using 27-plex Luminex assay. CF samples and healthy control samples were analyzed on different plates. Means ± SD of extrapolated values are presented. Values out of range below were set to 1.0. IL-10, and leptin was out of range below for both CF and HC BALF samples. Spearman correlation between cytokines measured in CF BALF (*n* = 7) with LDH releases to CM at 1- or 5-h stimulation. BALF, bronchoalveolar lavage fluid; CF, cystic fibrosis; CM, conditioned medium; HC, healthy control subjects; LDH, lactate dehydrogenase; SD, standard deviation.

**Table 5.** Correlations of parameters in BALF with LDH

	LDH CM 1 h		LDH CM 5 h	
Parameter in BALF	Spearman *r*	*P* value	Spearman *r*	*P* value
Total protein	−0.108	0.711	0.495	0.074
Osmolality	−0.018	0.951	0.506	0.056
dsDNA	0.151	0.627	0.370	0.174
Histones	0.595	0.132	0.467	0.248
Protease	0.667	0.267	0.600	0.350

Spearman correlation between different parameters (total protein, osmolality, dsDNA, histones, and protease) measured in BALF (patients with CF and healthy control samples) with LDH releases to CM at 1- or 5-h stimulation. BALF, bronchoalveolar lavage fluid; CF, cystic fibrosis; CM, conditioned medium; LDH, lactate dehydrogenase.

#### Aspergillus+ BALF significantly increases hMSC death.

To further explore potential causes of hMSC death, the microbiological status of the CF BALF was assessed. No correlation was observed with Staphylococcus or Pseudomonas bacterial infections (Table 6**)**. However, we noted that CF BALF samples that cultured positive for *Aspergillus* species (Asp+), predominantly *A. fumigatus*, demonstrated that the Asp+ but not Asp− samples induced cell death. Although there was some variability between individual samples, 1-h hMSC exposure to BALF from Asp+ patients with CF, compared with BALF from HC subjects, elicited a significant increase in LDH release (*P* = 0.028) (Fig. 1*D*). Notably, exposure to Asp− CF BALF did not provoke increased LDH release. These differences were even more marked following 5 h of exposure (*P* = 0.014) (Fig. 1*E*). Comparisons of total protein, osmolality, dsDNA content, histone concentration, protease activity, and levels of pro- and anti-inflammatory cytokines revealed no significant differences between Asp+ and Asp− BALF.

**Table 6.** Bacteria detected in CF BALF samples did not correlate with LDH release

	LDH CM 1 h		LDH CM 5 h		Detection Rate
Bacteria detected in CF BALF	*R^2^*	*P* value	*R^2^*	*P* value	Number (%)
*Staphylococcus*	0	>0.999	0.058	0.929	5/11 (45%)
*Pseudomonas*	na	na	na	na	0/11 (0%)

Spearman correlation between bacteria detected in CF BALF samples (Staphylococcus and Pseudomonas) with LDH release to CM at 1- or 5-h stimulation. Positive culture of bacteria was set to 1, and negative results were set to 0. BALF, bronchoalveolar lavage fluid; CF, cystic fibrosis; CM, conditioned medium; LDH, lactate dehydrogenase; na, not available.

#### Gliotoxin induces hMSC death.

**Fig. 3.**
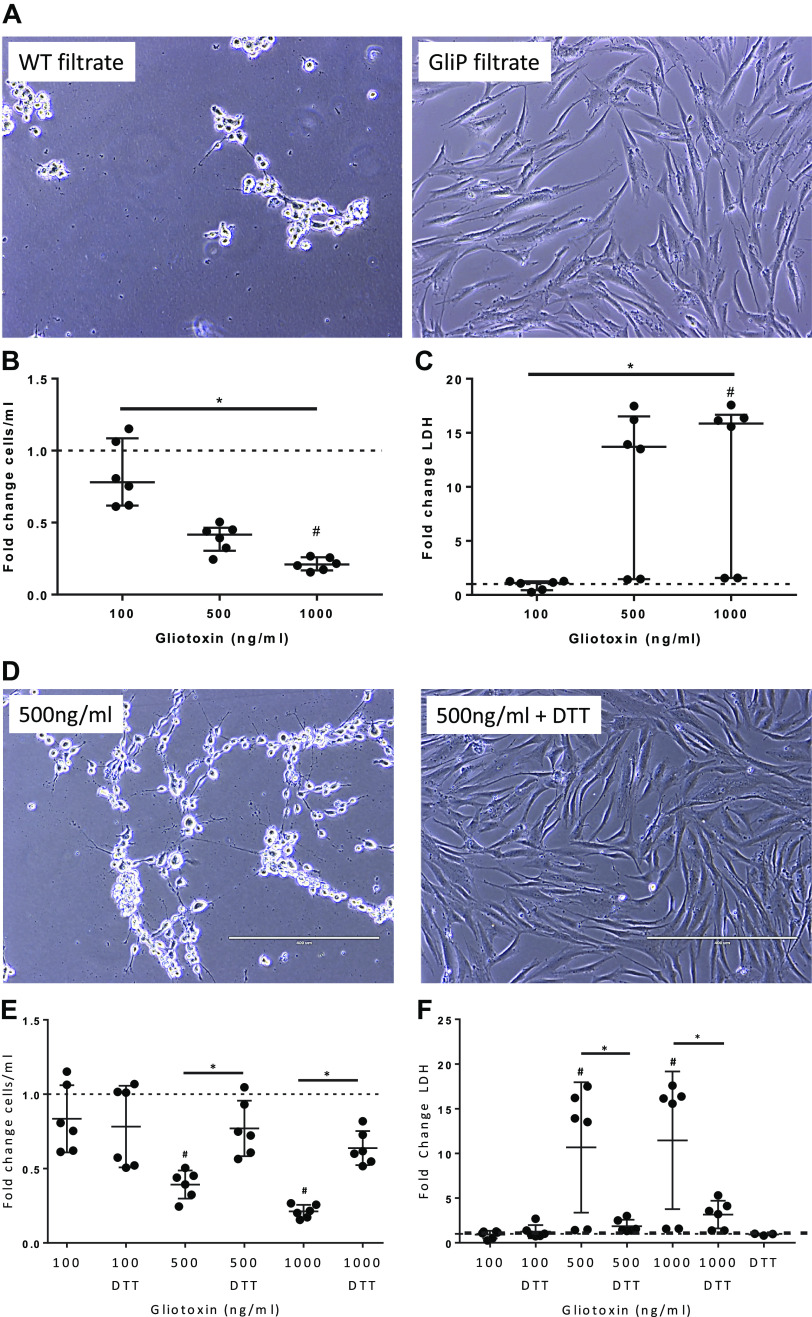

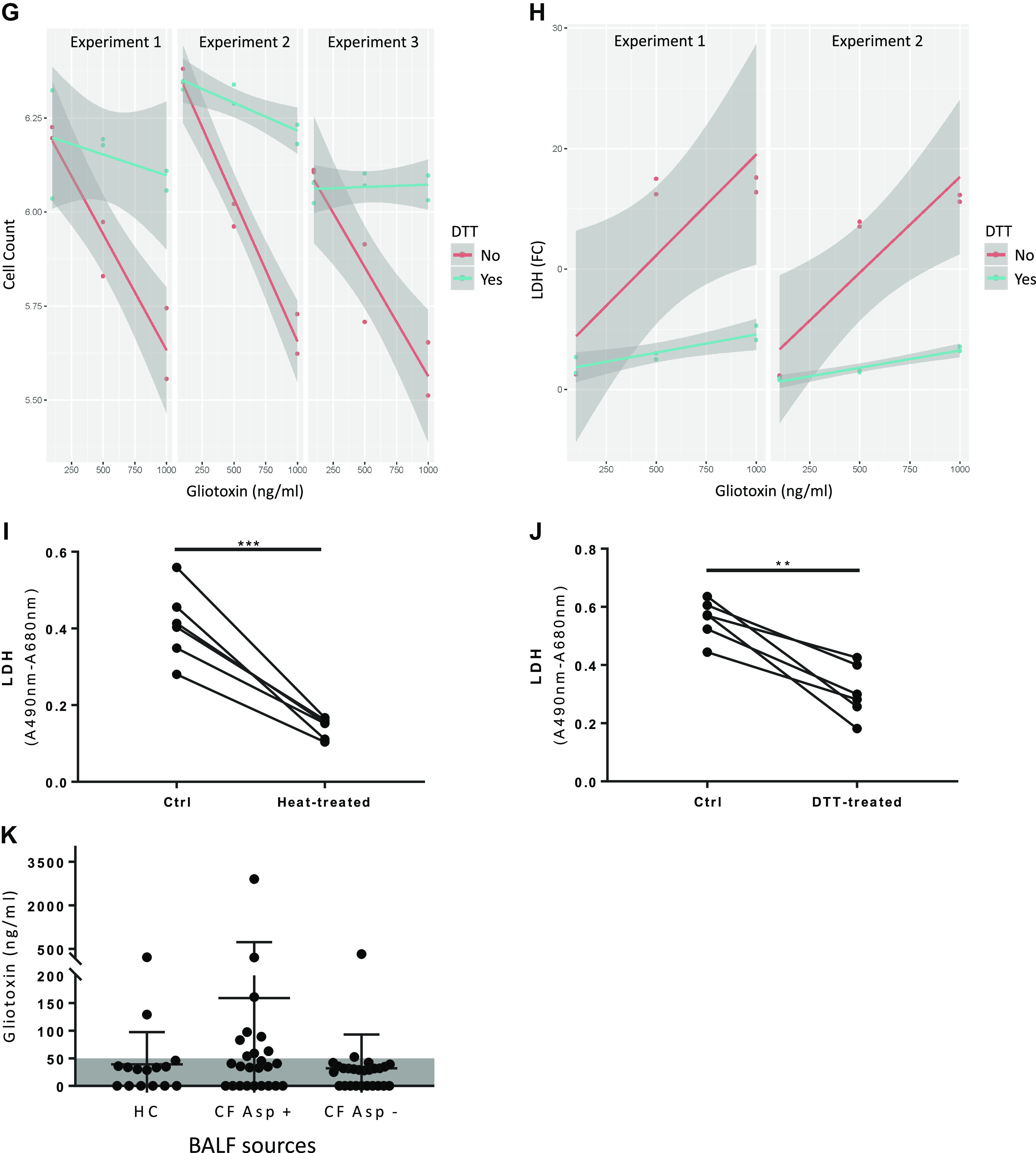
Gliotoxin mediates *Aspergillus*-induced hMSC toxicity. hMSCs exposed to wild-type *Aspergillus* filtrate (WT filtrate) but not mutant ΔgliP filtrate (no gliotoxin production) for 1 h exhibited significant cell death (*A*). Cell viability (*n* = 6 for each experimental condition) was assessed using trypan blue dye counts and by standard LDH assay and demonstrated dose-dependent toxic effects (*B *and *C*). Data are presented as median with interquartile of fold change (gliotoxin treated/DMSO control treated) of combined data from three individual experiments with statistical analysis performed by nonparametric one-way ANOVA (Kruskal–Wallis) followed by Dunn’s post hoc test. Gliotoxin was reconstituted in DMSO that was added to the unstimulated as a vehicle control (same volume as the highest gliotoxin concentration). A representative photomicrograph demonstrates that exposure to purified gliotoxin (500 ng/mL) for 5 h induced significant hMSC death, which was prevented by 100 µM DTT (diluted in PBS) (*D*). Scale bar represents 400 µm. Reduction of pure gliotoxin by addition of DTT inhibited the hMSC killing effect (*n* = 6 for each experimental condition, except for DTT only with *n* = 3), which was demonstrated by trypan blue cell count (*E*) and LDH released into the conditioned media (*F*). Data are presented as means ± SD of fold change of combined data from three individual experiments with statistical analysis performed by one-way ANOVA followed by Tukey’s post hoc test. Gliotoxin was reconstituted in DMSO, which was added to the unstimulated as a vehicle control (same volume as the highest gliotoxin concentration). Linear regression modeling of gliotoxin and DTT effects on cell count (three experiments) and LDH release (two experiments) are depicted in (*G *and* H*). Heat treatment (92.4°C–95°C for 20 min, *n* = 6 for each experimental condition) (*I*) or addition of DTT (100 µM, *n* = 6 for each experimental condition) (*J*) to Asp+ CF BALF decreases cytotoxicity as measured by LDH release in conditioned medium. Data are presented as means ± SD of combined data from three to four individual experiments with statistical analysis performed by paired t test. DTT-treated BALF samples were normalized to DTT controls. BALF gliotoxin levels are depicted in (*K*) with data presented as means ± SD with statistical analysis performed by linear regression modeling (HC: *n* = 15, CF Asp+: *n* = 25, CF Asp−: *n* = 26). Asp+, *Aspergillus* positive; Asp−, *Aspergillus* negative; BALF, bronchoalveolar lavage fluid; ctrl, untreated BALF control sample; DTT, dithiothreitol; HC, healthy volunteers; hMSC, human bone marrow-derived mesenchymal stromal cell; LDH, lactate dehydrogenase; *n* = number of samples. ***P ≤ 0.001. Photomicrographs have been brightness/contrast adjusted.

Gliotoxin is one of the major mycotoxins produced and secreted by *Aspergillus* species (10). To assess whether gliotoxin might play a role in hMSC death, cells were exposed to conditioned media (filtrate) obtained from a wild-type *Aspergillus fumigatus* strain (AF293, WT filtrate) and a gliotoxin null mutant *Aspergillus* strain (Δ*gliP*) (10). Media from the WT strain killed the majority of hMSCs within 1 h of exposure, but cells exposed to media from the Δ*gliP* strain appeared normal (Fig. 3*A*), suggesting that gliotoxin induces hMSC death.

Identification of gliotoxin as one of the likely mediators responsible for the observed hMSC death was further supported by exposing hMSCs to pure gliotoxin (Fig. 3*B*). Consistent with the WT filtrate results, a decrease in cell viability and increased LDH release were observed in a dose-dependent manner in hMSC cultures exposed to 500 ng/mL (*P* = 0.376 and *P* = 0.090, respectively) or 1,000 ng/mL (*P* = 0.013 and *P* = 0.029, respectively) gliotoxin, compared with unstimulated control hMSCs (Fig. 3, *B* and *C*). Reduction of disulfide bridges in gliotoxin by addition of dithiothreitol (DTT), a well-recognized method of neutralizing gliotoxin (46), inhibited the killing effect (3, *D*–*F*) demonstrating specificity. In Fig. 3*F*, two outliers were observed, but it was unclear if this was biological or assay variability. To assess this further, these two data points were removed, and corresponding linear regression analyses were performed on the remaining samples. This analysis also indicated that gliotoxin increased cell death (reduced cell count, *P* = 2.56E^−14^, linear model gliotoxin main effect) and that gliotoxin and DTT were strongly antagonistic (*P* = 2.41E^−9^, linear model interaction between gliotoxin and DTT) (Fig. 3*G*). Increased LDH release in gliotoxin-exposed hMSC cultures was also significantly blocked by DTT (*P* = 0.0004, linear model interaction between gliotoxin and DTT) (Fig. 3*H*).

To further determine whether the killing effect observed with Asp+ CF BALF was mediated by gliotoxin, the BALF samples were either heat-inactivated or DTT-treated before hMSC exposure. Although neither method is completely specific for inhibition of gliotoxin, in accordance with the pure gliotoxin stimulation data, the Asp+ BALF-induced hMSC death was significantly inhibited by both methods (*P* = 0.001 and *P* = 0.002, respectively) (Fig. 3, *I* and *J*). A potential clinically relevant pathogenic effect of gliotoxin on hMSCs is further supported by the significantly increased probability of detecting gliotoxin in Asp+ CF BALF samples [36%, odds ratio (OR) = 5.06, *P* = 0.023], compared with Asp− CF BALF samples (7.7%) and HC BALF samples (13%). Quantitative assessment of gliotoxin concentrations in BALF by mass spectrometry demonstrated that, although there were outliers in each grouping, Asp+ BALF samples contained higher levels compared with Asp− or HC BALF samples (Fig. 3*K*). However, as the BALF dilution, and, thus, the actual in vivo gliotoxin concentration, is unknown for each sample, the outliers and the differences in concentrations across the Asp+ CF samples suggest that gliotoxin may be one of several factors in Asp+ BALF inducing hMSC death.

#### Gliotoxin-induced hMSC cell death depends partly on mitochondrial damage.

**Fig. 4.**
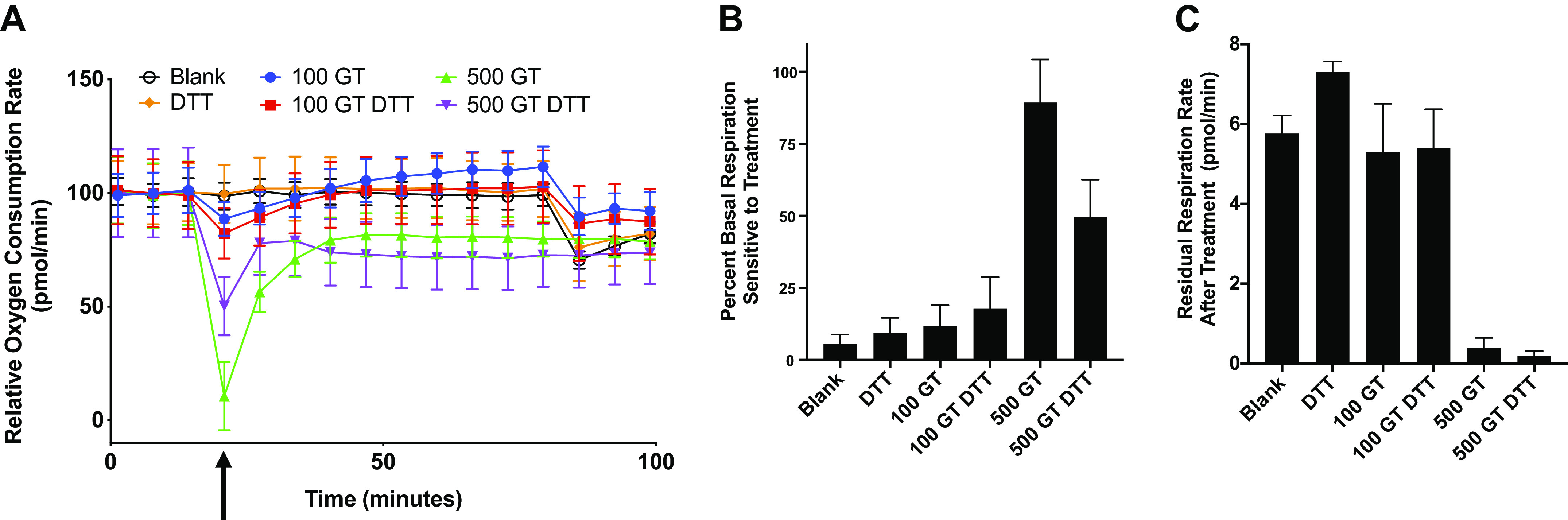
Gliotoxin-induced MSC cell death depends partly on mitochondrial damage. hMSCs were plated at a density of 75,000 cells/well in XF-96^e^ cell culture plate (Agilent Seahorse Bioscience). Basal respiration was measured, following injection of indicated treatment (XF Media, DTT alone, 100 ng/mL of gliotoxin, 100 ng/mL of gliotoxin + DTT, 500 ng/mL of gliotoxin, 500 ng/mL of gliotoxin + DTT). Measurements continued for 1 h, and then, a second injection of 100-nM rotenone/antimycin-A was introduced. Normalized percent rate changes from baseline are depicted (*A*). Percent basal respiration sensitive to treatment was calculated as the difference between the basal respiration rate and the respiration rate immediately following treatment injection (*B*). Residual respiration rate after treatment was calculated as the difference between the posttreatment respiration rate and the average respiration rate following rotenone/antimycin-A injection (*C*). For all graphs, means ± SE of at least four replicate wells and data are representative of at least three independent experiments. Blank, XF media control; DTT, dithiothreitol; GT, gliotoxin; hMSC, human bone marrow-derived mesenchymal stromal cell; MSC, mesenchymal stromal cell. Arrow indicates treatment addition between the third and fourth time points.

The exact mechanism through which gliotoxin induces cell death has not been fully elucidated; however, it has been suggested that mycotoxins, such as gliotoxin, induce mitochondrial dysfunction (33). To assess the impact of gliotoxin on hMSC mitochondrial function, oxygen consumption rate (OCR) was measured in real time on gliotoxin-exposed hMSCs using an XF-96^e^ Extracellular Flux Analyzer. Gliotoxin (500 ng/mL) rapidly decreased OCR within several minutes (Fig. 4*A*). This reduction was partly (50%) inhibited by DTT (Fig. 4, *A* and *B*). Notably, 1 h following 500 ng/mL gliotoxin exposure, hMSCs had almost no intact nonmitochondrial oxygen consumption (Fig. 4*C*). In accordance with the cytotoxicity data, the effect of lower doses of gliotoxin (100 ng/mL) had a marginal effect on OCR (Fig. 4). These data suggest that dose-dependent mitochondrial dysfunction rapidly occurs following hMSC exposure to gliotoxin.

#### hMSCs exposed to CF versus HC BALF have profound differences in gene expression profile.

**Fig. 5.**
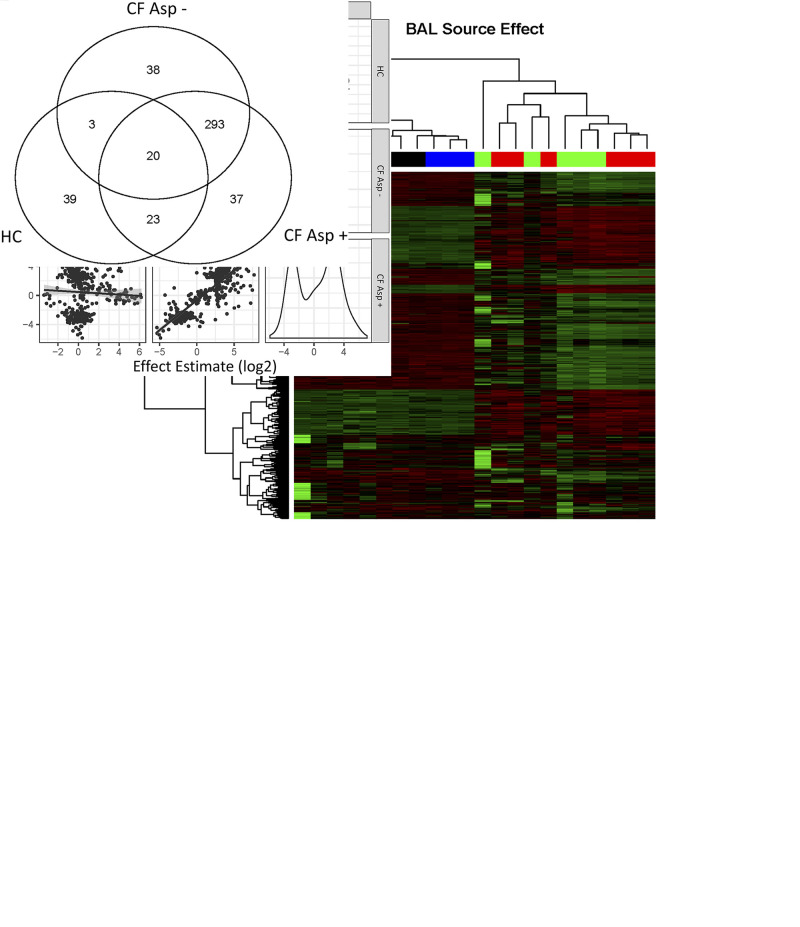
Profound differences were observed in the gene expression profile of hMSCs exposed to CF BALF and healthy controls. Unsupervised clustering of Z scored, normalized gene expression values from 453 genes that differed significantly (FDR < 0.05, edgeR) from unstimulated controls shows that CF BALF samples are substantially similar [CF Asp+ (red): *n* = 6, or CF Asp− (green): *n* = 5] but different from unstimulated hMSCs (blue, *n* = 5) and hMSCs exposed to healthy control (HC, black, *n* = 6) BALF samples (*A *and* B*). Z-scores measure difference in standard units from the mean for each gene, where green color indicates higher expression and red color indicates lower expression. The Venn diagram represents the number of genes in each category whose expression differed significantly from unstimulated based on edgeR analysis, FDR < 0.05 (*C*). Asp+, *Aspergillus* positive; Asp−, *Aspergillus* negative; BALF, bronchoalveolar lavage fluid; CF, cystic fibrosis; FDR, false discovery rate; HC, healthy volunteers; hMSC, human bone marrow-derived mesenchymal stromal cell.

To ascertain the effects of Asp+ BALF on hMSC gene expression, RNA sequencing was used to assess early changes in gene expression of hMSCs exposed to Asp+ or Asp− CF or to HC BALF. Unsupervised hierarchical clustering analyses of the 453 genes that were differentially expressed (edgeR: FDR < 0.05) compared with HC show that normalized expression values of differentially expressed genes in the HC cluster together with unstimulated samples on the left and samples from CF Asp+ and CF Asp− cluster together on the right (Fig. 5*A*). These data demonstrate that, although present, the differences between CF Asp+ and CF Asp− appear to be smaller than the differences between CF samples and HC samples. To assess this further, levels of correlation between effects estimated for exposure to BALF from CF Asp+ and CF Asp− and HC were compared with unstimulated controls. As shown in Fig. 5*B*, log2 fold changes in CF Asp+ and CF Asp− correlated very well, with a Pearson r of 0.856. On the other hand, CF effects did not correlate well with HC effects, with Pearson r values less than 0.2. Nonetheless, slightly different sets of genes were significantly differentially expressed (FDR < 0.05, edgeR) in response to CF Asp+ and CF Asp− BALF (Fig. 5*C*). Taken together, these results are consistent with the hypothesis that different inflammatory lung environments differentially alter hMSC gene expression, with the largest differences being attributable to whether BALF came from HC or CF sources.

#### hMSCs exposed to Aspergillus+ CF BALF samples have altered expression of genes associated with antimicrobial defense, interferon signaling, and cell death.

**Fig. 6.**
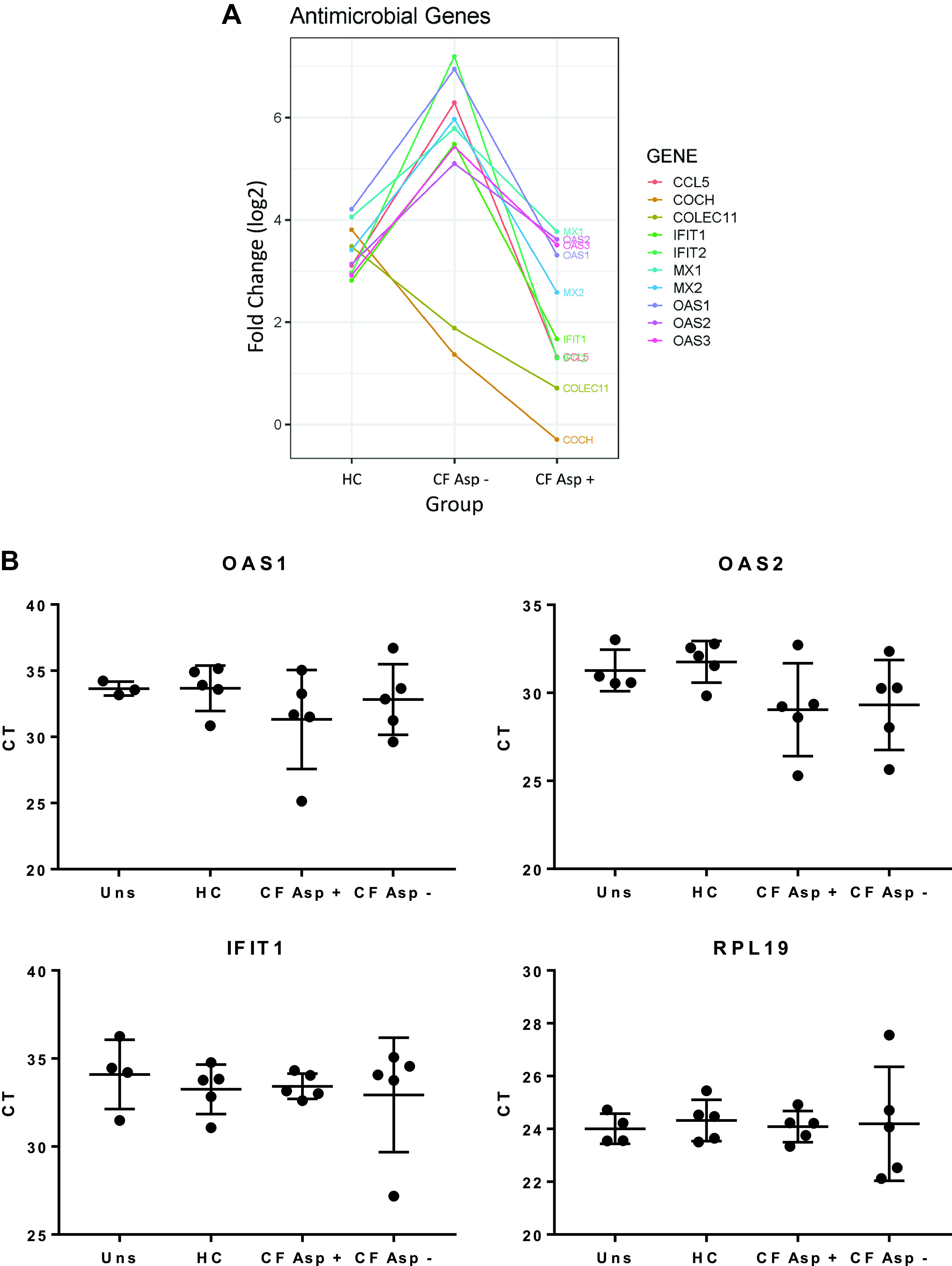
Exposure of hMSCs to Asp+ CF versus Asp− CF BALF has differential effects on expression of genes associated with antimicrobial defense. Informatics assessments of the RNA sequencing data (Ingenuity) demonstrated differential effects on relevant hMSC behaviors including expression of antimicrobial genes (*A*). *y*-axis is log2 fold change compared with unstimulated. Verification of the antimicrobial genes OAS1, OAS2, and IFIT1 using RT-PCR (uns: *n* = 3–4, HC: *n* = 5, CF Asp+: *n* = 5, CF Asp−: *n* = 5) (*B*). Data are presented as means ± SD of CT values with statistical analysis performed by one-way ANOVA followed by Tukey’s post hoc test. Asp+, *Aspergillus* positive; Asp−, *Aspergillus* negative; BALF, bronchoalveolar lavage fluid; CF, cystic fibrosis; HC, healthy volunteers; hMSC, human bone marrow-derived mesenchymal stromal cell; IFIT1, interferon-induced protein with tetratricopeptide repeats 1; OAS1, 2′-5′-oligoadenylate synthetase 1; OAS2, 2′-5′-oligoadenylate synthetase 2.

hMSCs exposed to Asp− CF BALF demonstrated increased expression of *CCL5*, *IFIT1*, *IFIT2*, *MX1*, *MX2*, *OAS1*, *OAS2*, and *OAS3*, genes all involved in antimicrobial defenses, compared with HC BALF-exposed hMSCs. However, in hMSCs exposed to Asp+ CF BALF, expression of these genes was reduced to levels similar or below levels found in hMSCs exposed to HC BALF (Fig. 6*A*). Similar trends were seen in the RT-PCR data for *OAS1*, *OAS2*, and *IFIT1*; however, the differences did not reach significance (Fig. 6*B*). To further study the differences observed within the CF BALF groups, gene expression profiles of hMSCs exposed to Asp+ and Asp− CF BALF samples were compared. Notably, pronounced differences in the antimicrobial genes *CCL5*, *COCH*, *IFIT1*, *IFIT2*, *MX1*, *MX2*, *OAS1*, *OAS2*, and *OAS3* were observed (Fig. 6*A*).

**Fig. 7.**
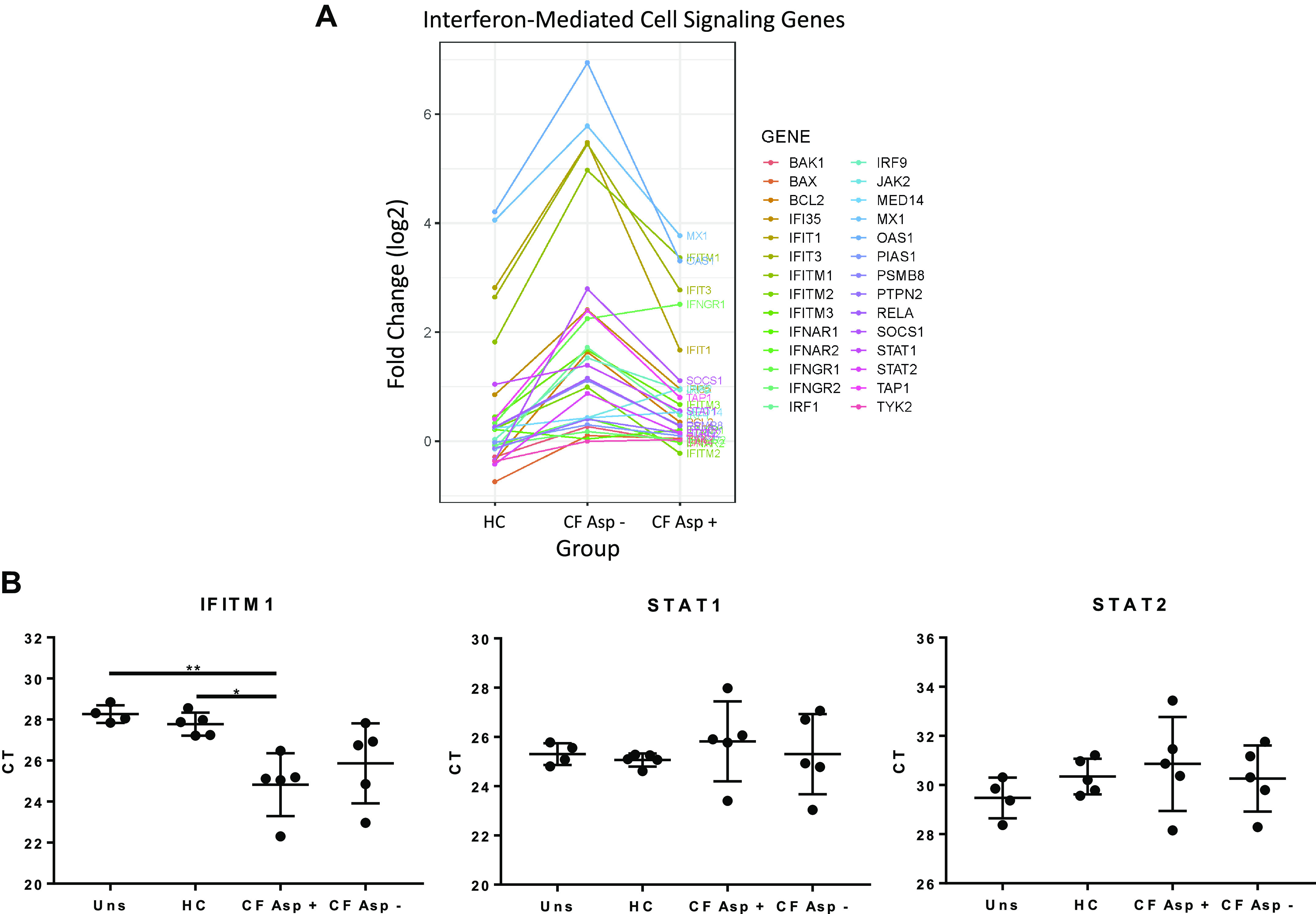
Exposure of hMSCs to Asp+ CF versus Asp− CF BALF has differential effects on expression of genes associated with interferon-mediated cell signaling. Informatics assessments of the RNA sequencing data (Ingenuity) demonstrated differential effects on relevant hMSC behavior including expression of interferon signaling genes (*A*). *y*-axis is log2 fold change compared with unstimulated. Verification of the genes IFITM1, STAT1, and STAT2 using RT-PCR (uns: *n* = 3–4, HC: *n* = 5, CF Asp+: *n* = 5, CF Asp-: *n* = 5) (*B*). Data are presented as means ± SD of CT values with statistical analysis performed by one-way ANOVA followed by Tukey’s post hoc test. Asp+, *Aspergillus* positive; Asp−, *Aspergillus* negative; BALF, bronchoalveolar lavage fluid; CF, cystic fibrosis; HC, healthy volunteers; hMSC, human bone marrow-derived mesenchymal stromal cell; IFITM1, interferon-induced transmembrane protein 1; STAT1, signal transducer and activator of transcription 1; STAT2, signal transducer and activator of transcription 2.

**Fig. 8.**
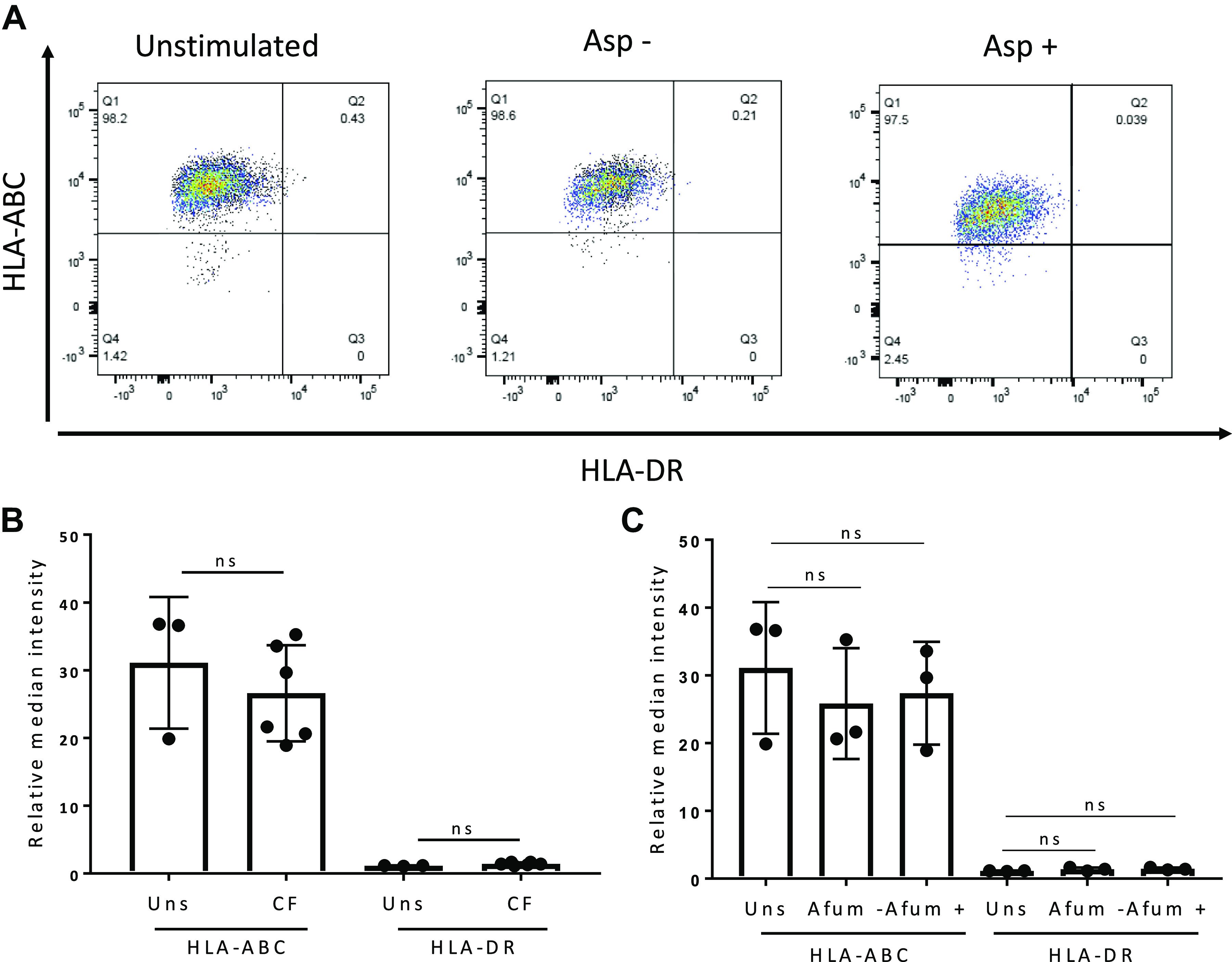
Exposure of hMSCs to CF BALF did not alter cell surface HLA-ABC or DR expression. HLA-ABC and HLA-DR surface marker expression for hMSCs following 5 h of exposure to CF BALF (*n* = 6 biological samples, two replicates per sample). Unstimulated (*n* = 3 experiments, two replicates per sample) hMSCs were used as controls (*A*). Relative median intensity of HLA-ABC and DR surface marker expression on unstimulated hMSCs and hMSCs exposed to CF BALF samples (*B*). Relative intensity of HLA-ABC and DR surface marker expression on unstimulated hMSCs (*n* = 3 experiments, two replicates per sample) and hMSCs exposed to Asp+ (*n* = 3 biological samples, two replicates per sample) and Asp− (*n* = 3 biological samples, two replicates per sample) CF BALF samples (*C*). Asp+, Aspergillus positive; Asp−, Aspergillus negative; BALF, bronchoalveolar lavage fluid; CF, cystic fibrosis; HC, healthy volunteers; hMSC, human bone marrow-derived mesenchymal stromal cell; ns, not statistically significant.

Similarly, expression of genes in the interferon-mediated cell signaling pathways, including *JAK2*, *SOCS1*, and *IFITM1*, was decreased in hMSCs exposed to Asp+ CF BALF compared with hMSCs exposed to Asp− CF BALF (Fig. 7*A*). Several genes in antimicrobial signaling pathways, notably *MX1*, *OAS1*, *IFIT1*, and *IFIT3*, differentially altered by exposure to Asp+ versus Asp− CF BALF have been shown to be important in IFN signaling. Also, a trend toward increase in *STAT1* gene expression was observed in all groups compared with unstimulated control cells; however, only hMSCs stimulated with CF Asp− BALF demonstrated a significant increase compared with unstimulated controls (*P* = 0.055). Similar trends were seen in the RT-PCR data for *STAT1* and *STAT2* (Fig. 7*B*). In addition, a trend toward increased *BCL-2*, *BAX*, and *BAK1* expression, genes known to be related to cell death, was observed in CF BALF-exposed hMSCs compared with hMSCs exposed to HC BALF and unstimulated control cells (Fig. 7*A*). Finally, hMSCs exposed to Asp+ CF BALF demonstrated significantly higher expression of *IFITM1* compared with hMSCs exposed to HC and unstimulated cells (*P* = 0.015 and *P* = 0.007, respectively) (Fig. 7*B*). Although altered expression of genes involved in interferon signaling was observed, no statistically significant differences were observed in HLA-ABC or DR surface marker expression in CF BALF-exposed, either Asp+ or Asp−, compared with unstimulated hMSCs (Fig. 8).

## DISCUSSION

These studies demonstrate that CF BALF obtained from lungs infected or colonized with *Aspergillus* can be cytotoxic to hMSCs. Our approach, using clinical BALF samples as a surrogate for the in vivo clinical environment, has been validated in recent work, including our own studies (1, 21, 32). The novel finding of this study is that Asp+ BALF samples obtained from patients with acute exacerbations of CF lung disease have significant effects on a number of relevant hMSC functions, including viability, IFN-mediated cell signaling, and antimicrobial gene expression. Determining hMSC behavior in the setting of *Aspergillus* lung infection may be critical as many patients with lung disease, who are otherwise candidates for potential hMSC-based therapies, may be colonized or infected with *Aspergillus*, with or without clinical manifestations such as allergic bronchopulmonary aspergillosis.

The observed adverse effects of CF BALF on hMSC appear to be mediated, at least in part, through *Aspergillus*-produced gliotoxin, acting initially to disrupt mitochondrial oxygen utilization. However, other as-yet unidentified mycotoxins, fungal proteases, and other mediators may also be involved (8). The presence of gliotoxin in lung tissue and in serum has been reported in both experimentally induced invasive aspergillosis and human aspergillosis, as well as in BALF samples from patients with CF (9, 27). However, the concentration of gliotoxin in lungs and in BALF needs to be better defined for different clinical conditions, including CF. Clinical studies of aspergillosis in patients with CF are often confounded by lack of specificity in fungal identification and characterization of individual strains, and our study faced similar issues. For example, gliotoxin was detected in two of the HC BALF samples and two of the Asp− BALF samples with no documented *Aspergillus* lung infection, even though none of these samples induced hMSC death. This illustrates the difficulties in culturing *Aspergillus* species even under optimal academic hospital clinical laboratory conditions and highlights that otherwise healthy controls can be colonized with *Aspergillus* species. It is also known that not all *Aspergillus* species produce gliotoxin. Although the majority of *Aspergillus* samples cultured from the BALF samples were *A. fumigatus*, it is not always possible to determine the precise *Aspergillus* species from the clinical BALF samples. Furthermore, there are several known isoforms of gliotoxin, including a nontoxic form, bis(methylthio)gliotoxin, and the detection method used in this study could not discriminate between the isoforms (14). This is likely to explain the absence of a clear correlation between BALF gliotoxin concentration and LDH release by the exposed hMSCs in a clinical study of this scale. We are currently attempting to better clarify these issues, including assessing a larger number of CF BALF samples with and without *Aspergillus* colonization/infection to better delineate differences in gliotoxin and other potential mediators of hMSC death. This also includes more in-depth comparative analyses of pro- and anti-inflammatory cytokine content and other inflammatory mediators such as neutrophil elastase not measured with the analytical approach used. One additional potential approach is suggested by the observation that gliotoxin synthesis is encoded by a multigene cluster including several genes that neutralize gliotoxin intracellularly to protect *Aspergillus* itself from toxicity (41). In a notable study, transducing yeast to express one of these protective genes subsequently protected the yeast against gliotoxin toxicity (44). A similar approach could conceivably be undertaken with the hMSCs.

The RNA sequencing data demonstrated alterations of IFN-mediated cell signaling pathways in hMSCs exposed to Asp+ BALF samples compared with unstimulated and HC. These are relevant findings, as IFN-γ-mediated hMSC activation is well recognized in other systems and has been extensively investigated for priming MSCs (7, 22, 50). For example, IFN-γ-pretreated MSCs reduced the symptoms and increased the survival rate of graft-versus-host disease in NOD-SCID mice compared with untreated MSCs (22). In addition, several studies have reported that IFN-γ-treated MSCs upregulate MHC class I and II expression (17, 26). However, we did not detect any statistically significant differences in HLA-ABC or DR surface marker expression on BALF-exposed hMSCs after 5-h stimulation (Fig. 8). However, longer time points might be required to see changes in the transcriptional regulation of self- and antigen-presenting genes.

Another relevant finding was that the RNA sequencing data demonstrated differential expression of antimicrobial genes, an important potential mechanism by which hMSCs might be protective in CF lungs. Increases in expression of a number of the antimicrobial genes, including *CCL5* and *COLEC1*, were observed in hMSC exposure to Asp− CF BALF. However, this was not observed in hMSCs exposed to Asp+ CF BALF samples. *CCL5* is a chemokine that plays an important role in inflammation by recruiting inflammatory cells, including T cells and macrophages (29). Interestingly, *CCL5* has been demonstrated to be dysregulated in peripheral blood mononuclear cells in plasma from patients with CF (20). *COLEC1* is a protein that belongs to the collectin family and plays an important role in the innate immune system. *COLEC1* binds to carbohydrate structures on and induces lysis of pathogens such as bacteria and fungi (47). Notably, insufficient amount of mannose-binding lectin has been associated with adverse progression of CF lung disease (18). Taken together, microbes and the chronic inflammatory environment present in CF lungs have an important impact on hMSC functions. Comparable findings have recently been demonstrated in patients with ARDS, where the inflammatory environment elicits an anti-inflammatory response in hMSCs (21, 32).

RNA sequencing data also demonstrated that gliotoxin induced the expression of genes involved in apoptotic pathways, including *BAX* and *BAK1*. These are relevant findings, as gliotoxin from *Aspergillus* has been suggested to induce *BIM/BAK*-dependent apoptosis through JNK-dependent phosphorylation (19). One potential explanation for not observing a robust activation of genes that mediate cell death in the RNA sequencing experiment could be that cells were only exposed for 1 h, whereas the majority cell death was observed after gliotoxin exposures of 5 h.

Interestingly, we have previously demonstrated that systemic administration of bone marrow-derived MSCs of both mouse and human origin as well as extracellular vesicles derived from the MSCs decreased allergic airway inflammation in mice resulting from mucosal immunization with commercially available *Aspergillus* hyphal extract (11–13, 24). However, it is presently unknown whether the hyphal extract contained gliotoxin, and we are exploring this further. We have also found only one other manuscript with respect to MSCs and *Aspergillus*. Schmidt et al. (40) found in in vitro studies that exposure of human MSCs to *A. fumigatus* hyphae increased mRNA but not protein levels of IL-6 in MSCs. The MSCs were able to phagocyte *Aspergillus* conidia but had no subsequent alteration in cytokine production. The MSCs were not adversely affected by exposure to either hyphae or conidia, but it is not clear if either contained gliotoxin. There is only limited other available data on interaction of MSCs with other fungi. Arango et al. (3) found that MSC administration worsened paracoccidioidomycosis infection and corresponding inflammation in a mouse model. The same group has now recently demonstrated that the *Paracoccidioides* fungus can activate MSCs via TLR2, TLR4, and dectin engagement (37).

In conclusion, these data underscore the importance of considering the in vivo inflammatory environment when using MSC-based cell therapy approaches for patients with inflammatory or infectious lung diseases such as fungal infections with *Aspergillus*. Conceivably, innovative approaches to preconditioning or engineering hMSCs to resist deleterious effects of the inflammatory environment encountered might help to enhance potential therapeutic effects (15, 16). Further focus has been on systemic MSC administration, and it is currently unknown whether direct airway MSC administration may also be altered by the presence of *Aspergillus* or other fungal infections. This is also an important area for future study.

## GRANTS

The RNA sequencing was performed by Dr. Fred W. Kolling at the Geisel School of Medicine at Dartmouth in the Genomics Shared Resources, which was established by equipment grants from the National Institutes of Health and National Science Foundation and is supported by a Cancer Center Core Grant (P30CA023108) from the National Cancer Institute. The flow cytometry data were performed at the Harry Hood Bassett Flow Cytometry and Cell Sorting Facility (FACS) at UVM. We acknowledge assistance from the NHLBI PACT program, University of Minnesota, Molecular and Cellular Therapeutics (contract HHSN268201000008). The authors also acknowledge the European Union (EU) and European Respiratory Society (ERS) for financial support.

## DISCLOSURES

No conflicts of interest, financial or otherwise, are declared by the authors.

## AUTHOR CONTRIBUTIONS

S.C.A., T.H.H., R.A.C., B.A.S., M.J.W., D.J.W., and S.R.E. conceived and designed research; S.C.A., E.H., J.D., K.S.S., D.E.M., E.A., J.B., and S.R.E. performed experiments; S.C.A., E.H., J.D., T.H.H., K.S.S., D.E.M., E.A., and S.R.E. analyzed data; S.C.A., T.H.H., E.H., J.D., A.K., K.E., B.M., C.d.S., F.F.C., P.R.M.R., K.D.L., M.A.M., R.A.C., B.A.S., M.J.W., D.J.W., and S.R.E. interpreted results of experiments; T.H.H., E.A., and S.R.E. prepared figures; D.J.W. and S.R.E. drafted manuscript; S.C.A., E.H., J.D., T.H.H., K.S.S., D.E.M., E.A., J.B., A.A., D.H.M., D.C.C., A.K., K.E., B.M., C.d.S., F.F.C., P.R.M.R., K.D.L., M.A.M., R.A.C., B.A.S. M.J.W., D.J.W., and S.R.E. edited and revised manuscript; S.C.A., E.H., J.D., T.H.H., K.S.S., D.E.M., E.A., J.B., A.A., D.H.M., D.C.C., A.K., K.E., B.M., C.d.S., F.F.C., P.R.M.R., K.D.L., M.A.M., R.A.C., B.A.S., M.J.W., D.J.W., and S.R.E. approved final version of manuscript.
